# Why we habitually engage in null-hypothesis significance testing: A qualitative study

**DOI:** 10.1371/journal.pone.0258330

**Published:** 2021-10-15

**Authors:** Jonah Stunt, Leonie van Grootel, Lex Bouter, David Trafimow, Trynke Hoekstra, Michiel de Boer

**Affiliations:** 1 Department of Health Sciences, Section of Methodology and Applied Statistics, Vrije Universiteit, Amsterdam, The Netherlands; 2 Department of Radiation Oncology, Erasmus Medical Center, Rotterdam, The Netherlands; 3 Rathenau Institute, The Hague, The Netherlands; 4 Department of Philosophy, Vrije Universiteit, Amsterdam, The Netherlands; 5 Department of Epidemiology and Data Science, Amsterdam University Medical Centers, Amsterdam, The Netherlands; 6 Psychology Department, New Mexico State University, Las Cruces, New Mexico, United States of America; 7 Department of General Practice and Elderly Care, University Medical Center Groningen, Groningen, The Netherlands; University of British Columbia, CANADA

## Abstract

**Background:**

Null Hypothesis Significance Testing (NHST) is the most familiar statistical procedure for making inferences about population effects. Important problems associated with this method have been addressed and various alternatives that overcome these problems have been developed. Despite its many well-documented drawbacks, NHST remains the prevailing method for drawing conclusions from data. Reasons for this have been insufficiently investigated. Therefore, the aim of our study was to explore the perceived barriers and facilitators related to the use of NHST and alternative statistical procedures among relevant stakeholders in the scientific system.

**Methods:**

Individual semi-structured interviews and focus groups were conducted with junior and senior researchers, lecturers in statistics, editors of scientific journals and program leaders of funding agencies. During the focus groups, important themes that emerged from the interviews were discussed. Data analysis was performed using the constant comparison method, allowing emerging (sub)themes to be fully explored. A theory substantiating the prevailing use of NHST was developed based on the main themes and subthemes we identified.

**Results:**

Twenty-nine interviews and six focus groups were conducted. Several interrelated facilitators and barriers associated with the use of NHST and alternative statistical procedures were identified. These factors were subsumed under three main themes: the scientific climate, scientific duty, and reactivity. As a result of the factors, most participants feel dependent in their actions upon others, have become reactive, and await action and initiatives from others. This may explain why NHST is still the standard and ubiquitously used by almost everyone involved.

**Conclusion:**

Our findings demonstrate how perceived barriers to shift away from NHST set a high threshold for actual behavioral change and create a circle of interdependency between stakeholders. By taking small steps it should be possible to decrease the scientific community’s strong dependence on NHST and p-values.

## Introduction

Empirical studies often start from the idea that there might be an association between a specific factor and a certain outcome within a population. This idea is referred to as the alternative hypothesis (H1). Its complement, the null hypothesis (H0), typically assumes no association or effect (although it is possible to test other effect sizes than no effect with the null hypothesis). At the stage of data-analysis, the probability of obtaining the observed, or a more extreme, association is calculated under the assumption of no effect in the population (H0) and a number of inferential assumptions [1]. The probability of obtaining the observed, or more extreme, data is known as ‘the p-value’. The p-value demonstrates the compatibility between the observed data and the expected data under the null hypothesis, where 0 is complete incompatibility and 1 is perfect compatibility [2]. When the p-value is smaller than a prespecified value (labelled as alpha, usually set at 5% (0.05)), results are generally declared to be statistically significant. At this point, researchers commonly reject the null hypothesis and accept the alternative hypothesis [2]. Assessing statistical significance by means of contrasting the data with the null hypothesis is called Null Hypothesis Significance Testing (NHST). NHST is the best known and most widely used statistical procedure for making inferences about population effects. The procedure has become the prevailing paradigm in empirical science [3], and reaching and being able to report statistically significant results has become the ultimate goal for many researchers.

Despite its widespread use, NHST and the p-value have been criticized since its inception. Numerous publications have addressed problems associated with NHST and p-values. Arguably the most important drawback is the fact that NHST is a form of indirect or inverse inference: researchers usually want to know if the null or alternative hypothesis can be accepted and use NHST to conclude either way. But with NHST, the probability of a finding, or more extreme findings, *given the null hypothesis* is calculated [4]. Ergo, NHST doesn’t tell us what we want to know. In fact, p-values were never meant to serve as a basis to draw conclusions, but as a continuous measure of incompatibility between empirical findings and a statistical model [2]. Moreover, the procedure promotes a dichotomous way of thinking, by using the outcome of a significance test as a dichotomous indicator for an effect (p<0.05: effect, p>0.05: no effect). Reducing empirical findings to two categories also results in a great loss of information. Further, a significant outcome is often unjustly interpreted as relevant, but a p-value does not convey any information about the strength or importance of the association. Worse yet, the p-values on which NHST is based confound effect size and sample size. A trivial effect size may nevertheless result in statistical significance provided a sufficiently large sample size. Or an important effect size may fail to result in statistical significance if the sample size is too small. P-values do not validly index the size, relevance, or precision of an effect [5]. Furthermore, statistical models include not only null hypotheses, but additional assumptions, some of which are wrong, such as the ubiquitous assumption of random and independent sampling from a defined population [1]. Therefore, although p-values validly index the incompatibility of data with models, p-values do not validly index incompatibility of data with hypotheses that are embedded in wrong models. These are important drawbacks rendering NHST unsuitable as the default procedure for drawing conclusions from empirical data [2,3,5–13].

A number of alternatives have been developed that overcome these pitfalls, such as Bayesian inference methods [7,11,14,15], informative hypothesis testing [9,16] and a priori inferential statistics [4,17]. These alternatives build on the idea that research usually starts with a more informed research-question than one merely assuming the null hypothesis of no effect. These methods overcome the problem of inverse inference, although the first two might still lead to dichotomous thinking with the use of thresholds. Despite the availability of alternatives, statistical behavior in the research community has hardly changed. Researchers have been slow to adopt alternative methods and NHST is still the prevailing paradigm for making inferences about population effects [3].

Until now, reasons for the continuous and ubiquitous use of NHST and the p-value have scarcely been investigated. One explanation is that NHST provides a very simple means for drawing conclusions from empirical data, usually based on the 5% cut-off. Secondly, most researchers are unaware of the pitfalls of NHST; it has been shown that NHST and the p-value are often misunderstood and misinterpreted [2,3,8,11,18,19]. Thirdly, NHST has a central role in most methods and statistics courses in higher education. Courses on alternative methods are increasingly being offered but are usually not mandatory. To our knowledge, there is a lack of in depth, empirical research, aimed at elucidating why NHST nevertheless remains the dominant approach, or what actions can be taken to shift the sciences away from NHST. Therefore, the aim of our study was to explore the perceived barriers and facilitators, as well as behavioral intentions related to the use of NHST and alternatives statistical procedures, among all relevant stakeholders in the scientific system.

### Theoretical framework

**Fig 1:**
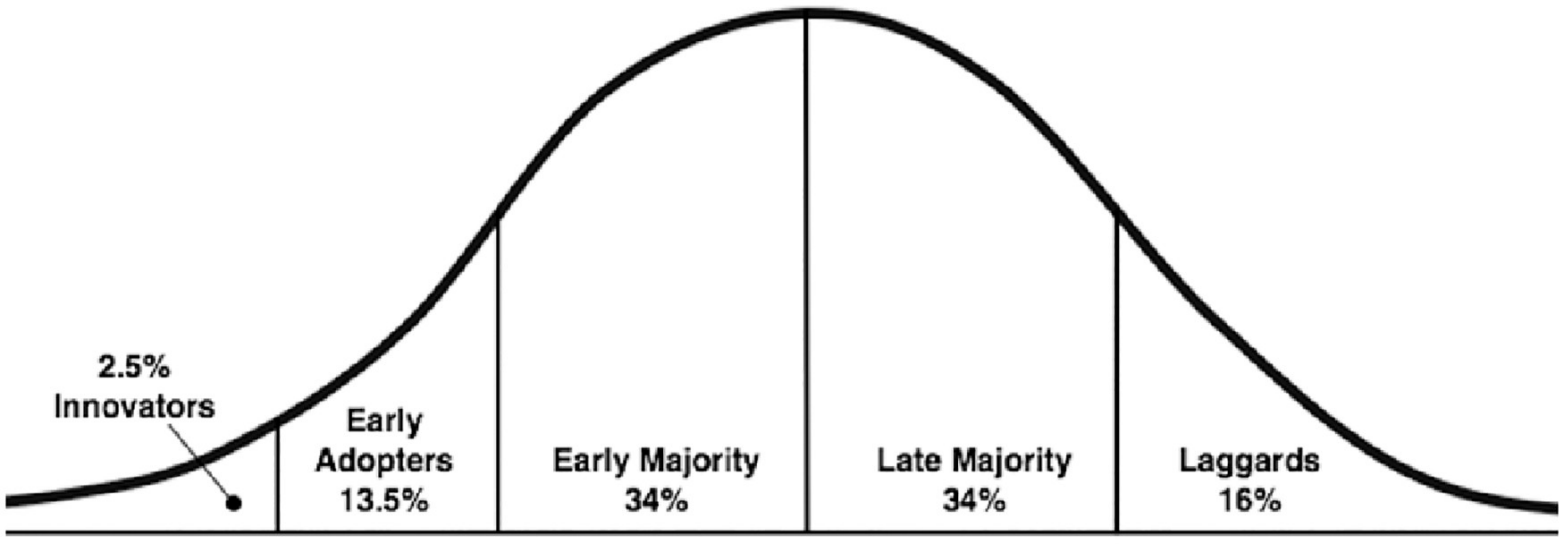
Adopter categorization. The innovativeness dimension, measured by the time at which an individual from an adopter category adopts an innovation. Each category is one of more standard deviations removed from the average time of adoption [20].

In designing our study, we used two theories. Firstly, we used the ‘diffusion of innovation theory’ of Rogers [20]. This theory describes the dissemination of an innovation as a process consisting of four elements: 1) an innovation is 2) communicated through certain channels 3) over time 4) among the members of a social system [20]. In the current study, the innovation consists of the idea that we should stop with the default use of NHST and instead consider using alternative methods for drawing conclusions from empirical data. The science system forms the social structure in which the innovation should take place. The most important members, and potential adopters of the innovation, we identified are researchers, lecturers, editors of scientific journals and representatives of funding agencies. Rogers describes phases in the adoption process, which coincide with characteristics of the (potential) adopters of the idea: 1) innovators, 2) early adopters, 3) early majority adopters, 4) late majority adopters and 5) laggards. Innovators are the first to adopt an innovation. There are few innovators but these few are very important for bringing in new ideas. Early adopters form the second group to adopt an innovation. This group includes opinion leaders and role models for other stakeholders. The largest group consists of the early and late majority who follow the early adopters, and then there is a smaller group of laggards who resist the innovation until they are certain the innovation will not fail. The process of innovation adoption by individuals is described as a normal distribution (Fig 1). For these five groups, the adoption of a new idea is influenced by the following five characteristics of the innovative idea and 1) its relative advantage, 2) its compatibility with current experiences, 3) its complexity, 4) its flexibility, and 5) its visibility [20]. Members of all four stakeholder groups could play an important role in the diffusion of the innovation of replacing NHST by its alternatives.

**Fig 2:**
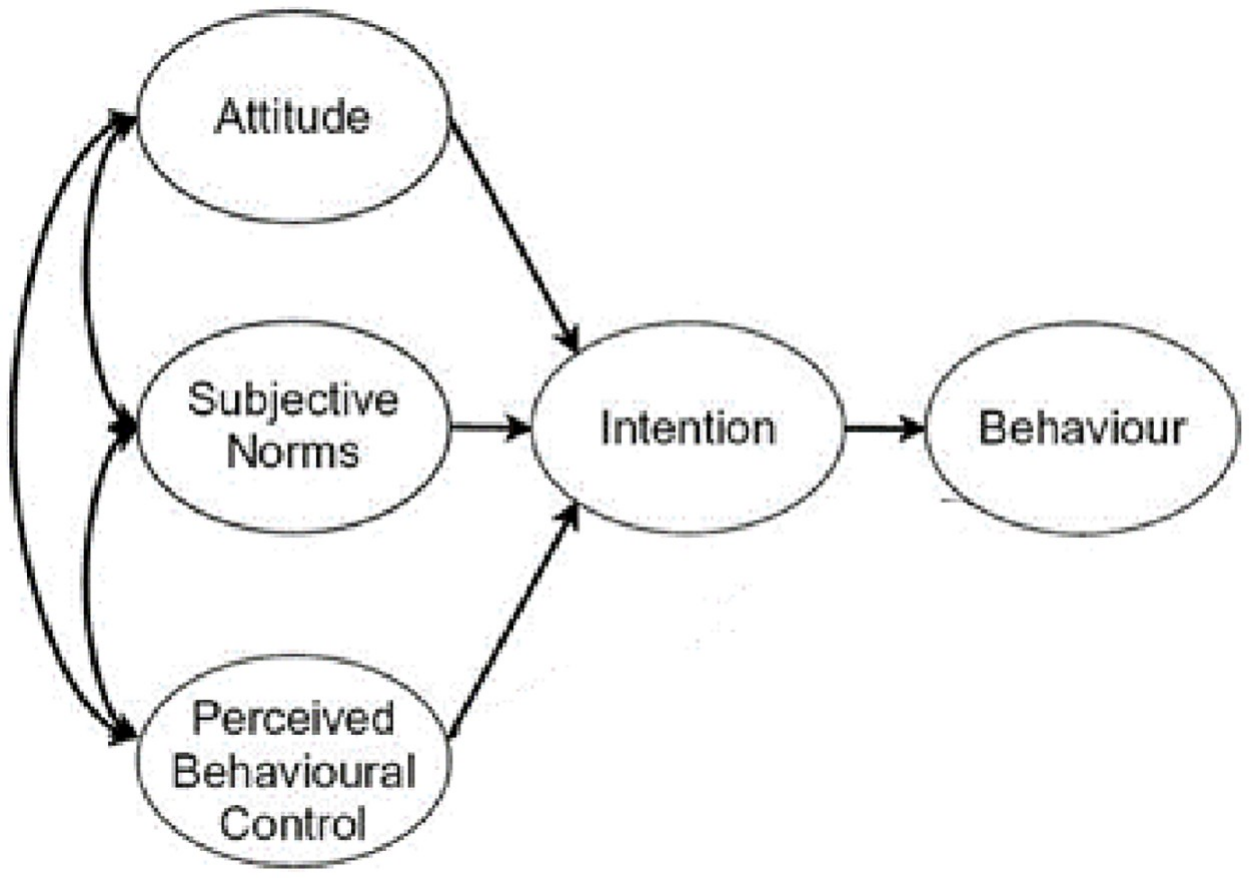
Theory of planned behavior [21].

Another important theory for our study is the ‘theory of planned behavior’, that was developed in the 1960s [21]. This theory describes how human behavior in a certain context can be predicted and explained. The theory was updated in 2010, under the name ‘the reasoned action approach’ [22]. A central factor in this theory is the intention to perform a certain behavior, in this case, to change the default use of NHST. According to the theory, people’s intentions determine their behaviors. An intention indexes to what extent someone is motivated to perform the behavior. Intentions are determined by three independent determinants: the person’s attitudes toward the behavior—the degree to which a person sees the behavior as favorable or unfavorable, perceived subjective norms regarding the behavior—the perceived social pressure to perform the behavior or not, and perceptions of control regarding the behavior—the perceived ease or difficulty of performing the behavior. Underlying (i.e. responsible for) these three constructs are corresponding behavioral, normative, and control beliefs [21,22] (see Fig 2).

Both theories have served as a lens for both data collection and analysis. We used sensitizing concepts [23] within the framework of the grounded theory approach [24] from both theories as a starting point for this qualitative study, and more specifically, for the topic list for the interviews and focus groups, providing direction and guidance for the data collection and data analysis.

Many of the concepts of Rogers’ and Fishbein and Ajzen’s theory can be seen as facilitators and barriers for embracing and implementing innovation in the scientific system.

## Methods

### Design

A qualitative study among stakeholders using semi-structured interviews and focus groups was performed. Data collection and analysis were guided by the principle of constant comparison traditional to the grounded theory approach we followed [24]. The grounded theory is a methodology that uses inductive reasoning, and aims to construct a theory through the collection and analysis of data. Constant comparison is the iterative process whereby each part of the data that emerges from the data analysis is compared with other parts of the data to thoroughly explore and validate the data. Concepts that have been extracted from the data are tagged with codes that are grouped into categories. These categories constitute themes, which (may) become the basis for a new theory. Data collection and analysis were continued until no new information was gained and data saturation had likely occurred within the identified themes.

### Sample

The target population consisted of stakeholders relevant to our topic: junior and senior researchers, lecturers in statistics, editors of scientific journals and program leaders of funding agencies (see Tables 1 and 2). We approached participants in the field of medical sciences, health- and life sciences and psychology. In line with the grounded theory approach, theoretical sampling was used to identify and recruit eligible participants. Theoretical sampling is a form of purposive sampling. This means that we aimed to purposefully select participants, based on their characteristics that fit the parameters of the research questions [25]. Recruitment took place by approaching persons in our professional networks and or the networks of the approached persons.

**Table 1:** General overview of participants.

Stakeholder group:		
	*Total*	*Male*: *female*
**Researcher**	13	6:7
**Lecturer**	15	8:7
**Editor**	11	5:6
**Representative of funding agency**	8	3:5

**Table 2:** Characteristics of the participants split up by interviews and focus groups.

Stakeholder group:	Workplace:				
	**Interviews**				**total**
	***University***	***Research institute***	***Funding agency***	***Academic hospital***	
**Researcher**	2		1	1	4
**Lecturer**	9				9
**Editor**	4			4	8
**Representative of funding agency**			8		8
	**Focus groups**				
	***University***	***Research institute***	***Funding agency***	***Academic hospital***	
**Researcher**	5	1		3 (1)	9
**Lecturer**	9 (3*)			4 (2)	13
**Editor**	2 (1)			1	3
**Representative of funding agency**			2 (2)		2

*The numbers between brackets represents the number of participants that were also interviewed.

### Data collection

We conducted individual semi-structured interviews followed by focus groups. The aim of the interviews was to gain insight into the views of participants on the use of NHST and alternative methods and to examine potential barriers and facilitators related to these methods. The aim of the focus groups was to validate and further explore interview findings and to develop a comprehensive understanding of participants’ views and beliefs.

#### Interviews

For the semi-structured interviews, we used a topic list (see Appendix 1 in S1 Appendix). Questions addressed participants’ knowledge and beliefs about the concept of NHST, their familiarity with NHST, perceived attractiveness and drawbacks of the use of NHST, knowledge of the current NHST debate, knowledge of and views on alternative procedures and their views on the future of NHST. The topic list was slightly adjusted based on the interviews with editors and representatives from funding agencies (compared to the topic list for interviews with researchers and lecturers). Questions particularly focused on research and education were replaced by questions focused on policy (see Appendix 1 in S1 Appendix).

The interviews were conducted between October 2017 and June 2018 by two researchers (L.v.G. and J.S.), both trained in qualitative research methods. Interviews lasted about one hour (range 31–86 minutes) and were voice-recorded. One interview was conducted by telephone; all others were face to face and took place at a location convenient for the participants, in most cases the participants’ work location.

#### Focus groups

During the focus groups, important themes that emerged from the interviews were discussed and explored. These include perceptions on NHST and alternatives and essential conditions to shift away from the default use of NHST.

Five focus groups included representatives from the different stakeholder groups. One focus group was homogenous, including solely lecturers. The focus groups consisted of ‘old’ as well as ‘new’ participants, that is, some of the participants of the focus groups were also in the interview sample. We also selected persons that were open for further contribution to the NHST debate and were willing to help think about (implementing) alternatives for NHST.

The focus groups were conducted between September and December 2018 by three researchers (L.v.G., J.S. and A.d.K.), all trained in qualitative research methods. The focus groups lasted about one-and-a-half hours (range 86–100 minutes).

#### Data analysis

All interviews and focus groups were transcribed verbatim. Atlas.ti 8.0 software was used for data management and analysis. All transcripts were read thoroughly several times to identify meaningful and relevant text fragments and analyzed by two researchers (J.S. and L.v.G.). Deductive predefined themes and theoretical concepts were used to guide the development of the topic list for the semi-structured interviews and focus groups, and were used as sensitizing concepts [23] in data collection and data analysis. Inductive themes were identified during the interview process and analysis of the data [26].

Transcripts were open-, axial- and selectively coded by two researchers (J.S. and L.v.G.). Open coding is the first step in the data-analysis, whereby phenomena found in the text are identified and named (coded). With axial coding, connections between codes are drawn. Selective coding is the process of selecting one central category and relating all other categories to that category, capturing the essence of the research. The constant comparison method [27] was applied allowing emerging (sub)themes to be fully explored. First, the two researchers independently developed a set of initial codes. Subsequently, findings were discussed until consensus was reached. Codes were then grouped into categories that were covered under subthemes, belonging to main themes. Finally, a theory substantiating the prevailing use of NHST was developed based on the main themes and subthemes.

#### Ethical issues

This research was conducted in accordance with the Dutch "General Data Protection Regulation" and the “Netherland’s code of conduct for research integrity”. The research protocol had been submitted for review and approved by the ethical review committee of the VU Faculty of Behavioral and Movement Sciences. In addition, the project had been submitted to the Medical Ethics Committee (METC) of the Amsterdam University Medical Centre who decided that the project is not subject to the *Medical Research (Human Subjects) Act (*WMO). At the start of data collection, all participants signed an informed consent form.

A full study protocol, including a detailed data analysis plan, was preregistered (https://osf.io/4qg38/). At the start of this study, preregistration forms for qualitative studies were not developed yet. Therefore, preregistration for this study is based on an outdated form. Presently, there is a preregistration form available for qualitative studies [28]. Information about data collection, data management, data sharing and data storage is described in a Data Management Plan. Sensitive data is stored in Darkstor, an offline archive for storing sensitive information or data (information that involves i.e., privacy or copyright). As the recordings and transcripts of the interviews and focus groups contain privacy-sensitive data, these files are archived in Darkstor and can be accessed only on request by authorized individuals (i.e., the original researcher or a research coordinator) (Data requests can be send to rdm@vu.nl). Non-sensitive data is stored in DANS (https://doi.org/10.17026/dans-2at-nzfs) (Data Archiving and Networked Services; the Netherlands institute for permanent access to digital research resources).

## Results

### Participant characteristics

Twenty-nine individual interviews and six focus groups were conducted. The focus groups included four to six participants per session. A total of 47 participants were included in the study (13 researchers, 15 lecturers, 11 editors of scientific journals and 8 representatives of funding agencies). Twenty-nine participants were interviewed. Twenty-seven participants took part in the focus group. Nine of the twenty-seven participants were both interviewed *and* took part in the focus groups. Some participants had multiple roles (i.e., editor and researcher, editor and lecturer or lecturer and researcher) but were classified based on their primary role (assistant professors were classified as lecturers). The lecturers in statistics in our sample were not statisticians themselves. Although they all received training in statistics, they were primarily trained as psychologists, medical doctors, or health scientists. Some lecturers in our sample taught an applied subject, with statistics as part of it. Other lectures taught Methodology and Statistics courses. Statistical skills and knowledge among lecturers varied from modest to quite advanced. Statistical skills and knowledge among participants from the other stakeholder groups varied from poor to quite advanced. All participants were working in the Netherlands. A general overview of the participants is presented in Table 1. Participant characteristics split up by interviews and focus groups are presented in Table 2.

### The model

**Fig 3:**
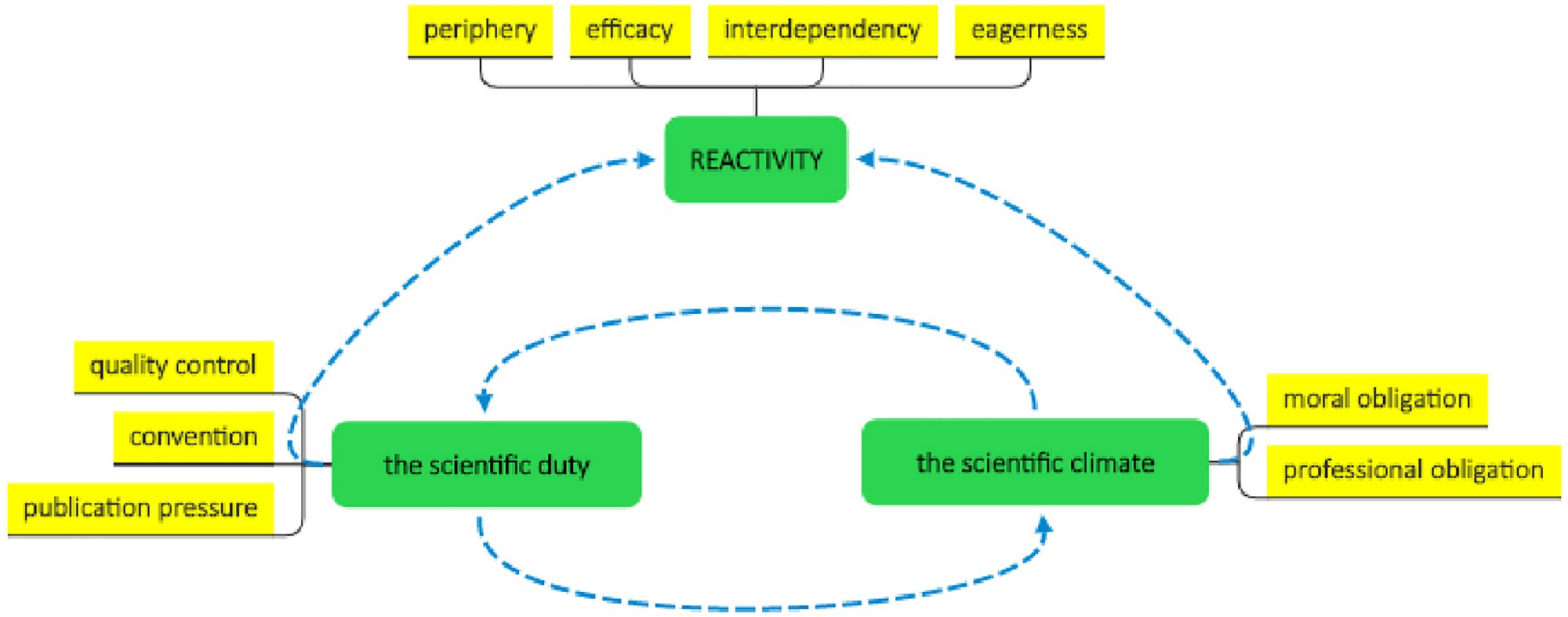
The coding scheme, representing the main and subthemes and how they are interrelated.

Three main themes with sub-themes and categories emerged (Fig 3): the green-colored compartments hold the three main themes: *The scientific climate*, *The scientific duty* and *Reactivity*. Each of these three main themes consists of subthemes, depicted by the yellow-colored compartments. In turn, some (but not all) of the 9 subthemes also have categories. These ‘lower level’ findings are not included in the figure but will be mentioned in the elaboration on the findings and are depicted in Appendix 2 in S1 Appendix. Fig 3 shows how the themes are related to each other. The blue arrows indicate that the themes are interrelated; factors influence each other. The scientific climate affects the way stakeholders perceive and fulfil their scientific duty, the way stakeholders give substance to their scientific duty shapes and maintain the scientific climate. The scientific duty and the scientific climate cause a state of reactivity. Many participants have adopted a ’wait and see’ attitude regarding behavioral changes with respect to statistical methods. They feel dependent on someone else’s action. This leads to a reactive (instead of a proactive) attitude and a low sense of responsibility. ‘Reactivity’ is the core theme, explaining the most critical problem with respect to the continuous and ubiquitous use of NHST.

Main themes and subthemes are numbered. Categories are mentioned in the body of the text in bold. ‘P’ stands for participant; ‘I’ stands for interviewer.

#### 1. The scientific climate

The theme, ‘the scientific climate’, represents researchers’ (Dutch) perceptions of the many written and unwritten rules they face in the research environment. This theme concerns the opportunities and challenges participants encounter when working in the science system. Dutch academics feel pressured to publish fast and regularly, and to follow conventions and directions of those on whom they depend. They feel this comes at the expense of the quality of their work. Thus, the scientific climate in the Netherlands has a strong influence on the behavior of participants regarding how they set their priorities and control the quality of their work.

*1*.*1 Quality control*. Monitoring the quality of research is considered very important. Researchers, funding agencies and editors indicate they rely on their own knowledge, expertise, and insight, and those of their colleagues, to guarantee this quality. However, editors or funding agencies are often left with little choice when it comes to compiling an evaluation committee or a review panel. The choice is often like-knows-like-based. Given the limited choice, they are forced to trust the opinion of their consultants, but the question is whether this is justified.

I: “The ones who evaluate the statistics, do they have sufficient statistical knowledge?” P: “Ehhr, no, I don’t think so.” I: “Okay, interesting. So, there are manuscripts published of which you afterwards might think….” P: “Yes yes.”

(Interview 18; Professor/editor, Medical Sciences)

*1*.*2 Convention*. The scientific system is built on mores and conventions, as this participant describes:

P: “There is science, and there is the sociology of science, that is, how we talk to each other, what we believe, how we connect. And at some point, it was agreed upon that we would talk to each other in this way.”

(Interview 28, researcher, Medical Sciences)

And to these conventions, one (naturally) conforms. Stakeholders copy behavior and actions of others within their discipline, thereby causing particular behaviors and values to become conventional or normative. One of those conventions is the use of NHST and p-values. Everyone is trained with NHST and is used to applying this method. Another convention is the fact that significant results mean ‘success’, in the sense of successful research and being a successful researcher. Everyone is aware that ‘p is smaller than 0.05’ means the desired results are achieved and that publication and citation chances are increased.

P: “You want to find a significant result so badly. (…) Because people constantly think: I must find a significant result, otherwise my study is worthless.”

(Focus group 4, lecturer, Medical Sciences)

Stakeholders rigidly hold on to the above-mentioned conventions and are not inclined to deviate from existing norms; they are, in other words, quite **conservative**. ‘We don’t know any better’ has been brought up as a valid argument by participants from various stakeholder groups to stick to current rules and conventions. Consequently, the status quo in the scientific system is being maintained.

P: “People hold on to….” I: ‘Everyone maintains the system?’ P: ‘Yes, we kind of hang to the conservative manner. This is what we know, what someone, everyone, accepts.”

(Interview 17, researcher, Health Sciences)

Everyone is trained with NHST and considers it an accessible and easy to interpret method. The familiarity and perceived simplicity of NHST, user-friendly software such as SPSS and the clear cut-off value for significance are important facilitators for the use of NHST and at the same time barriers to start using alternative methods. Applied researchers stressed the importance of the **accessibility** of NHST as a method to test hypotheses and draw conclusions. This accessibility also justifies the use of NHST when researchers want to communicate their study results and messages in understandable ways to their readership.

P: “It is harder, also to explain, to use an alternative. So, I think, but maybe I’m overstepping, but if you want to go in that direction [alternative methods] it needs to be better facilitated for researchers. Because at the moment… I did some research, but, you know, there are those uncommon statistical packages.”

(Interview 16, researcher/editor, Medical Sciences)

*1*.*3 Publication pressure*. Most researchers mentioned that they perceive publication pressure. This motivates them to use NHST and hope for significant results, as ‘significant p-values’ increase publication chances. They perceive a high workload and the way the scientific reward system is constructed as barriers for behavioral change pertaining to the use of statistical methods; potential negative consequences for publication and career chances prevent researchers from deviating from (un)written rules.

P: “I would like to learn it [alternative methods], but it might very well be that I will not be able to apply it, because I will not get my paper published. I find that quite tricky.”

(Interview 1, Assistant Professor, Health Sciences)

#### 2. The scientific duty

Throughout the interviews, participants reported a sense of duty in several variations. “What does it mean to be a scientific researcher?” seemed to be a question that was reflected upon during rather than prior to the interview, suggesting that many scientists had not really thought about the moral and professional obligations of being a scientist in general—let alone what that would mean for their use of NHST. Once they had given it some thought, the opinions concerning what constitutes the scientific duty varied to a large extent. Some participants attached great importance to issues such as reproducibility and transparency in scientific research and continuing education and training for researchers. For others, these topics seemed to play a less important role. A distinction was made between moral and professional obligations that participants described concerning their scientific duty.

*2*.*1 Moral obligation*. The moral obligation concerns issues such as doing research in a thorough and honest way, refraining from questionable research practices (QRPs) and investing in better research. It concerns tasks and activities that are not often rewarded or acknowledged.

Throughout the interviews and the focus groups, participants very frequently touched upon the responsibility they felt for doing ‘the right thing’ and making the right choice in doing research and using NHST, in particular. The extent to which they felt responsible varied among participants. When it comes to choices during doing research—for example, drawing conclusions from data—participants felt a strong sense of responsibility to do this correctly. However, when it comes to innovation and new practices, and feeling responsible for your own research, let alone improving scientific practice in general, opinions differed. This quotation from one of the focus groups illustrates that:

P1: “If you people [statisticians, methodologists] want me to improve the statistics I use in my research, then you have to hand it to me. I am not going to make any effort to improve that myself. “P3: “No. It is your responsibility as an academic to keep growing and learning and so, also to start familiarizing yourself when you notice that your statistics might need improvement.”

(Focus group 2, participant 1 (PhD researcher, Medical Sciences) and 3 (Associate Professor, Health Sciences)

The **sense of responsibility** for improving research practices regarding the use of NHST was strongly felt and emphasized by a small group of participants. They emphasized the responsibility of the researcher to think, interpret and be critical when interpreting the *p*-value in NHST. It was felt that you cannot leave that up to the reader. Moreover, scrutinizing and reflecting upon research results was considered a primary responsibility of a scientist, and failing to do so, as not living up to what your job demands you to do:

P: “Yes, and if I want to be very provocative—and I often want that, because then people tend to wake up and react: then I say that hiding behind alpha.05 is just scientific laziness. Actually, it is worse: it is scientific cowardice. I would even say it is ‘relieving yourself from your duty’, but that may sound a bit harsh…”

(Interview 2, Professor, Health Sciences)

These participants were convinced that scientists have a duty to keep scientific practice in general at the highest level possible.

The avoidance of **questionable research practices (QRPs)** was considered a means or a way to keep scientific practices high level and was often touched upon during the interviews and focus groups as being part of the scientific duty. Statisticians saw NHST as directly facilitating QRPs and providing ample examples of how the use of NHST leads to QRPs, whereas most applied researchers perceived NHST as the common way of doing research and were not aware of the risks related to QRPs. Participants did mention the violation of assumptions underlying NHST as being a QRP. Then, too, participants considered overinterpreting results as a QRP, including exaggerating the degree of significance. Although participants stated they were careful about interpreting and reporting p-values, they ‘admitted’ that statistical significance was a starting point for them. Most researchers indicated they search for information that could get their study published, which usually includes a low p-value (this also relates to the theme ‘Scientific climate’).

P: “We all know that a lot of weight is given to the p-value. So, if it is not significant, then that’s the end of it. If it ís significant, it just begins.”

(Interview 5, lecturer, Psychology)

The term ‘sloppy science’ was mentioned in relation to efforts by researchers to reduce the *p*-value (a.k.a. p-hacking, data-dredging, and HARKing. HARKing is an acronym that refers to the questionable research question of Hypothesizing After the Results are Known. It occurs when researchers formulate a hypothesis after the data have been collected and analyzed, but make it look like it is an a priori hypothesis [29]). Preregistration and replication were mentioned as being promising solutions for some of the problems caused by NHST.

*2*.*2*. *Professional obligation*. The theme professional obligation reflects participants’ expressions about what methodological knowledge scientists should have about NHST. In contrast moral obligations, there appeared to be some consensus about scientists’ professional obligations. Participants considered critical evaluation of research results a core professional obligation. Also, within all the stakeholder groups, participants agreed that sufficient statistical knowledge is required for using NHST, but they varied in their insights in the principles, potential and limitations of NHST. This also applied to the extent to which participants were aware of the current debate about NHST.

Participants considered **critical thinking** as a requirement for fulfilling their professional obligation. It specifically refers to the process of interpreting outcomes and taking all relevant contextual information into consideration. Critical thinking was not only literally referred to by participants, but also emerged by interpreting text fragments on the emphasis within their research. Researchers differed quite strongly in where the emphasis of their research outcomes should be put and what kind of information is required when reporting study results. Participants mentioned the proven effectiveness of a particular treatment, giving a summary of the research results, effect sizes, clinical relevance, p-values, or whether you have made a considerable contribution to science or society.

P: “I come back to the point where I said that people find it arbitrary to state that two points difference on a particular scale is relevant. They prefer to hide behind an alpha of 0.05, as if it is a God given truth, that it counts for one and for all. But it is just as well an invented concept and an invented guideline, an invented cut-off value, that isn’t more objective than other methods?”

(Interview 2, Professor, Health Sciences)

For some participants, especially those representing funding agencies, critical thinking was primarily seen as a prerequisite for the utility of the research. The focus, when formulating the research question and interpreting the results, should be on practical relevance and the contribution the research makes to society.

The term **‘ignorance’** arose in the context of the participants’ concern regarding the level of statistical knowledge scientists and other stakeholders have versus what knowledge they should have to adequately apply statistical analysis in their research. The more statistically competent respondents in the sample felt quite strongly about how problematic the lack of knowledge about NHST is among those who regularly use it in their research, let alone the lack of knowledge about alternative methods. They felt that regularly retraining yourself in research methods is an essential part of the professional obligation one has. Applied researchers in the sample agreed that a certain level of background knowledge on NHST was required to apply it properly to research and acknowledged their own ignorance. However, they had different opinions about what level of knowledge is required. Moreover, not all of them regarded it as part of their scientific duty to be informed about all ins and outs of NHST. Some saw it as the responsibility of statisticians to actively inform them (see also the subtheme periphery). Some participants were not aware of their ignorance or stated that some of their colleagues are not aware of their ignorance, i.e., that they are unconsciously incompetent and without realizing it, poorly understood what the p-value and associated outcome measures actually mean.

P: “The worst, and I honestly think that this is the most common, is unconsciously incompetent, people don’t even understand that…” I: “Ignorance.” P: “Yes, but worse, ignorant and not even knowing you are ignorant.”

(Interview 2, Professor, Health Sciences)

The lack of proper knowledge about statistical procedures was especially prevalent in the medical sciences. Participants working in or with the medical sciences all confirmed that there is little room for proper statistical training for medical students and that the level of knowledge is fairly low. NHST is often used because of its simplicity. It is especially attractive for medical PhD students because they need their PhD to get ahead in their medical career instead of pursuing a scientific career.

P: “I am not familiar with other ways of doing research. I would really like to learn, but I do not know where I could go. And I do not know whether there are better ways. So sometimes I do read studies of which I think: ‘this is something I could investigate with a completely different test. Apparently, this is also possible, but I don’t know how.’ Yes, there are courses, but I do not know what they are. And here in the medical center, a lot of research is done by medical doctors and these people have hardly been taught any statistics. Maybe they will get one or two statistics courses, they know how to do a t-test and that is about it. (…) And the courses have a very low level of statistics, so to say.”

(Interview 1, Assistant Professor, Health Sciences)

Also, the term ‘**awareness**’ arose. Firstly, it refers to being conscious about the limitations of NHST. Secondly, it refers to the awareness of the ongoing discussions about NHST and more broadly, about the replication crisis. The statisticians in the sample emphasized the importance of knowing that NHST has limitations and that it cannot be considered the holy grail of data analysis. They also emphasized the importance of being aware of the debate. A certain level of awareness was considered a necessary requirement for critical thinking. There was variation in that awareness. Some participants were quite informed and were also fairly engaged in the discussion whereas others were very new to the discussion and larger contextual factors, such as the replication crisis.

I: “Are you aware of the debate going on in academia on this topic [NHST]? P: “No, I occasionally see some article sent by a colleague passing by. I have the idea that something is going on, but I do not know how the debate is conducted and how advanced it is.

(Interview 6, lecturer, Psychology)

With respect to the theme, ‘the scientific duty’, participants differed to what extent they felt responsible for better and open science, for pioneering, for reviewing, and for growing and learning as a scientist. Participants had one commonality: although they strived for adherence to the norms of good research, the rampant feeling is that this is very difficult, due to the scientific climate. Consequently, participants perceive an **internal conflict**: a discrepancy between what they *want* or *believe*, and what they *do*. Participants often found themselves struggling with the responsibility they felt they had. Making the scientifically most solid choice was often difficult due to feasibility, time constraints, or certain expectations from supervisors (this is also directly related to the themes ‘Scientific climate’ and ‘Reactivity’). Thus, the scientific climate strongly influences the behavior of scientists regarding how they set their priorities and fulfill their scientific duties. The strong sense of scientific duty was perceived by some participants as a facilitator and by others as a barrier for the use of alternative methods.

#### 3. Reactivity

A consequence of the foregoing factors is that most stakeholders have adopted a reactive attitude and behave accordingly. People are disinclined to take responsibility and await external signals and initiatives of others. This might explain why NHST is being continuously used and remains the default procedure to make inferences about population effects.

The core theme ‘reactivity’ can be explained by the following subthemes and categories:

*3*.*1 Periphery*. The NHST-problem resides in the periphery in several ways. First, it is a subject that is not given much priority. Secondly, some applied researchers and editors believe that methodological knowledge, as it is not their field of expertise, should not be part of their job requirement. This also applies to the NHST debate. Thirdly, and partly related to the second point, there is a lack of cooperation within and between disciplines.

The term ‘**priority’** was mentioned often when participants were asked to what extent the topic of NHST was subject of discussion in their working environment. Participants indicated that (too) little priority is given to statistics and the problems related to the subject. There is simply a lot going on in their research field and daily work, so there are always more important or urgent issues on the agenda.

P: “Discussions take place in the periphery; many people find it complicated. Or are just a little too busy.”

(Interview 5, lecturer, Psychology)

As the NHST debate is not prioritized, initiatives with respect to this issue are not forthcoming. Moreover, researchers and lecturers claim there is neither time nor money available for training in statistics in general or acquiring more insight and skills with respect to (the use of) alternative methods. Busy working schedules were mentioned as an important barrier for improving statistical knowledge and skills.

P: “Well you can use your time once, so it is an issue low on the priority list.”

(Focus group 5, researcher, Medical Sciences)

The NHST debate is perceived as the domain of statisticians and methodologists. Also, cooperation between different domains and domain-specific experts is perceived as complicated, as different perceptions and ways of thinking can clash. Therefore, some participants feel that **separate worlds** should be kept separate; put another way: stick to what you know!

P: “This part is not our job. The editorial staff, we have the assignment to ensure that it is properly written down. But the discussion about that [alternatives], that is outside our territory.”

(Interview 26, editor, Medical Sciences)

Within disciplines, individuals tend to act on their own, not being aware that others are working on the same subject and that it would be worthwhile to join forces. The interviews and focus groups exposed that a modest number of participants actively try to change the current situation, but in doing that, feel like **lone voices** in the wilderness.

P1: “I mean, you become a lone voice in the wilderness.” P2: “Indeed, you don’t want that.” P1: “I get it, but no one listens. There is no audience.”

(Focus Group 3, P1: MD, lecturer, medical Sciences, P2: editor, Medical Sciences)

To succeed at positive change, participants emphasized that it is essential that people (interdisciplinary) cooperate and join forces, rather than operate on individual levels, focusing solely on their own working environment.

The caution people show with respect to taking initiative is reenforced by the fear of encountering resistance from their working environment when one voices that change regarding the use of NHST is needed. A condition that was mentioned as essential to bring about change was **tactical implementation**, that is, taking very small steps. As everyone is still using NHST, taking big steps brings the risk of losing especially the more conservative people along the way. Also, the adjustment of policy, guidelines and educational programs are processes for which we need to provide time and scope.

P: “Everyone still uses it, so I think we have to be more critical, and I think we have to look at some kind of culture change, that means that we are going to let go of it (NHST) more and we will also use other tests, that in the long term will overthrow NHST. I: and what about alternatives? P: I think you should never be too fanatic in those discussion, because then you will provoke resistance. (…) That is not how it works in communication. You will touch them on a sore spot, and they will think: ‘and who are you?’ I: “and what works?” P: “well, gradualness. Tell them to use NHST, do not burn it to the ground, you do not want to touch peoples work, because it is close to their hearts. Instead, you say: ‘try to do another test next to NHST’. Be a pioneer yourself.”

(Interview 5, lecturer, Psychology)

*3*.*2*. *Efficacy*. Most participants stated they feel they are not in the position to initiate change. On the one hand, this feeling is related to their hierarchical positions within their working environments. On the other hand, the feeling is caused by the fact that statistics is perceived as a very complex field of expertise and people feel they lack sufficient knowledge and skills, especially about alternative methods.

Many participants stated they felt little sense of empowerment, or self-efficacy. The academic system is perceived as hierarchical, having an unequal balance of power. Most participants believe that it is not in their power to take a lead in innovative actions or to stand up against establishment, and think that this responsibility lies with other stakeholders, that have more **status**.

P: “Ideally, there would be a kind of an emergency letter from several people whose names open up doors, in which they indicate that in the medical sciences we are throwing away money because research is not being interpreted properly. Well, if these people that we listen to send such an emergency letter to the board of The Netherlands Organization for Health Research and Development [the largest Dutch funding agency for innovation and research in healthcare], I can imagine that this will initiate a discussion.” (…) I: “and with a big name you mean someone from within the science system? P: well, you know, ideally a chairman, or chairmen of the academic medical center. At that level. If they would put a letter together. Yes, that of course would have way more impact. Or some prominent medical doctors, yes, that would have more impact, than if some other person would send a letter yes.”

(Interview 19, representative from funding agency, Physical Sciences)

Some participants indicated that they did try to make a difference but encountered too much resistance and therefore gave up their efforts. PhD students feel they have insufficient power to choose their own directions and make their own choices.

P: I am dependent on funding agencies and professors. In the end, I will write a grant application in that direction that gives me the greatest chance of eventually receiving that grant. Not primarily research that I think is the most optimal (…) If I know that reviewers believe the p-value is very important, well, of course I write down a method in which the p-value is central.”

(Focus group 2, PhD-student, Medical Sciences)

With a sense of imperturbability, most participants accept that they cannot really change anything.

Lastly, the **complexity** of the subject is an obstacle for behavioral change. Statistics is perceived as a difficult subject. Participants indicate that they have a lack of knowledge and skills and that they are unsure about their own abilities. This applies to the ‘standard’ statistical methods (NHST), but to a greater extent to alternative methods. Many participants feel that they do not have the capacity to pursue a true understanding of (alternative) statistical methods.

P: “Statistics is just very hard. Time and again, research demonstrates that scientists, even the smartest, have a hard time with statistics.”

(Focus group 3, PhD researcher, Psychology)

*3*.*3*. *Interdependency*. As mentioned, participants feel they are not in a sufficiently strong position to take initiative or to behave in an anti-establishment manner. Therefore, they await external signals from people within the scientific system with more status, power, or knowledge. This can be people within their own stakeholder group, or from other stakeholder groups. As a consequence of this attitude, a situation arises in which peoples’ actions largely depend on others. That is, a complex state of interdependency evolves: scientists argue that if the reward system does not change, they are not able to alter their statistical behavior. According to researchers, editors and funding agencies are still very much focused on NHST and especially (significant) p-values, and thus, scientists wait for editors and funders to adjust their policy regarding statistics:

P: “I wrote an article and submitted it to an internal medicine journal. I only mentioned confidence intervals. Then I was asked to also write down the p-values. So, I *had* to do that. This is how they [editors] can use their power. They decide.”

(Interview 1, Assistant Professor, Health Sciences)

Editors and funders in their turn claim they do not maintain a strict policy. Their main position is that scientists should reach consensus about the best statistical procedure, and they will then adjust their policy and guidelines.

P: “We actually believe that the research field itself should direct the quality of its research, and thus, also the discussions.”

(Interview 22, representative from funding agency, Neurosciences)

Lecturers, for their part, argue that they cannot revise their educational programs due to the academic system, and university policies are adapted to NHST and p-values.

As most participants seem not to be aware of this process, a circle of interdependency arises that is difficult to break.

P: “Yes, the stupid thing about this perpetual circle is that you are educating people, let’s say in the department of cardiology. They must of course grow, and so they need to publish. If you want to publish you must meet the norms and values of the cardiology journals, so they will write down all those p-values. These people are trained and in twenty years *they* are on the editorial board of those journals, and then you never get rid of it [the p-value].”

(Interview 18, Professor, editor, Medical Sciences)

*3*.*4*. *Degree of eagerness*. Exerting certain behavior or behavioral change is (partly) determined by the extent to which people *want* to employ particular behavior, their behavioral intention [22]. Some participants indicated they are willing to change their behavior regarding the use of statistical methods, but only if it is absolutely necessary, imposed or if they think that the current conventions have too many negative consequences. Thus, true, intrinsic will-power to change behavior is lacking among these participants. Instead, they have a rather **opportunistic** attitude, meaning that their behavior is mostly driven by circumstances, not by principles.

P: “If tomorrow an alternative is offered by people that make that call, than I will move along. But I am not the one calling the shots on this issue.”

(Interview 26, editor, Medical Sciences)

In addition, **pragmatism** often outweighs the perceived urgency to change. Participants argue they ‘just want to do their jobs’ and consider the practical consequences mainly in their actions. This attitude creates a certain degree of inertia. Although participants claim they are willing to change their behavior, this would contain much more than ‘doing their jobs, and thus, in the end, the NHST-debate is subject to ‘coffee talk’. People are open to discussion, but when it comes to taking action (and motivating others to do so), no one takes action.

P: “The endless analysis of your data to get something with a p-value less than 0.05… There are people that are more critical about that, and there are people that are less critical. But that is a subject for during the coffee break.”

(Interview 18, professor, editor, Medical Sciences)

## Discussion

The goal of our study was to acquire in-depth insight into reasons why so many stakeholders from the scientific system keep using NHST as the default method to draw conclusions, despite its many well-documented drawbacks. Furthermore, we wanted to gain insight into the reasons for their reluctance to apply alternative methods. Using a theoretical framework [20,21], several interrelated facilitators and barriers associated with the use of NHST and alternative methods were identified. The identified factors are subsumed under three main themes: the scientific climate, the scientific duty and reactivity. The scientific climate is dominated by conventions, behavioral rules, and beliefs, of which the use of NHST and p-values is part. At the same time, stakeholders feel they have a (moral or professional) duty. For many participants, these two sides of the same coin are incompatible, leading to internal conflicts. There is a discrepancy between what participants *want* and what they *do*. As a result of these factors, the majority feels dependent on others and have thereby become reactive. Most participants are not inclined to take responsibility themselves but await action and initiatives from others. This may explain why NHST is still the standard and used by almost everyone involved.

The current study is closely related to the longstanding debate regarding NHST which recently increased to a level not seen before. In 2015, the editors of the journal ‘Basic and Applied Social Psychology’ (BASP) prohibited the use of NHST (and p-values and confidence intervals) [30]. Subsequently, in 2016, the American Statistical Association published the so-called ‘Statement on p-values’ in the American Statistician. This statement consists of critical standpoints regarding the use of NHST and p-values and warns against the abuse of the procedure. In 2019, the American Statistician devoted an entire edition to the implementation of reforms regarding the use of NHST; in more than forty articles, scientists debated statistical significance, advocated to embrace uncertainty, and suggested alternatives such as the use of s-values, False Positive Risks, reporting results as effect sizes and confidence intervals and more holistic approaches to p-values and outcome measures [31]. In addition, in the same year, several articles appeared in which an appeal was made to stop using statistical significance testing [32,33]. A number of counter-reactions were published [34–36], stating (i.e.) that banning statistical significance and, with that, abandoning clear rules for statistical analyses may create new problems with regard to statistical interpretation, study interpretations and objectivity. Also, some methodologists expressed the view that under certain circumstances the use of NHST and p-values is not problematic and can in fact provide useful answers [37]. Until recently, the NHST-debate was limited to mainly methodologists and statisticians. However, a growing number of scientists are getting involved in this lively debate and believe that a paradigm shift is desirable or even necessary.

The aforementioned publications have constructively contributed to this debate. In fact, since the publication of the special edition of the American Statistician, numerous scientific journals published editorials or revised, to a greater or lesser extent, their author guidelines [38–45]. Furthermore, following the American Statistical Association (ASA), the National Institute of Statistical Sciences (NISS) in the United States has also taken up the reform issue. However, real changes are still barely visible. It takes a long time before these kinds of initiatives translate into behavioral changes, and the widespread adoption by most of the scientific community is still far from accomplished. Debate alone will not lead to real changes, and therefore, our efforts to elucidate behavioral barriers and facilitators could provide a framework for potential effective initiatives that could be taken to reduce the default use of NHST. In fact, the debate could counteract behavioral change. If there is no consensus among statisticians and methodologists (the innovators), changing behavior cannot be expected from stakeholders with less statistical and methodological expertise. In other words, without agreement among innovators, early adopters might be reluctant to adopt the innovation.

Research has recently been conducted to explore the potential of behavioral change to improve Open Science behaviors. The adoption of open science behavior has increased in the last years, but uptake has been slow, due to firm barriers such as a lack of awareness about the subject, concerns about constrainment of the creative process, worries about being “scooped” and holding on to existing working practices [46]. The development regarding open science practices and the parallels these lines of research shows with the current study, might be of benefit to subserve behavioral change regarding the use of statistical methods.

The described obstacles to change behavior are related to features of both the ‘innovative idea’ and the potential adopters of the idea. First, there are characteristics of ‘the innovation’ that form barriers. The first barrier is the complexity of the innovation: most participants perceive alternative methods as difficult to understand and to use. A second barrier concerns the feasibility of trying the innovation; most people do not feel flexible about trying out or experimenting with the new idea. There is a lack of time and monetary resources to get acquainted with alternative methods (for example, by following a course). Also, the possible negative consequences of the use of alternatives (lower publications chances, the chance that the statistical method and message is too complicated for one’s readership) is holding people back from experimenting with these alternatives. And lastly, it is unclear for most participants what the visibility of the results of the new idea are. Up until now, the debate has mainly taken place among a small group of statisticians and methodologists. Many researchers are still not aware of the NHST debate and the idea to shift away from NHST and use alternative methods instead. Therefore, the question is how easily the benefits of the innovation can be made visible for a larger part of the scientific community. Thus, our study shows that, although the compatibility of the innovation is largely consistent with existing values (participants are critical about (the use of) NHST and the p-value and believe that there are better alternatives to NHST), important attributes of the innovative idea negatively affect the rate of adoption and consequently the diffusion of the innovation.

Due to the barriers mentioned above, most stakeholders do not have the intention to change their behavior and adopt the innovative idea. From the theory of planned behavior [21], it is known that behavioral intentions directly relate to performances of behaviors. The strength of the intention is shaped by attitudes, subjective norms, and perceived power. If people evaluate the suggested behavior as positive (attitude), and if they think others want them to perform the behavior (subjective norm), this leads to a stronger intention to perform that behavior. When an individual also perceives they have enough control over the behavior, they are likely to perform it. Although most participants have a positive attitude towards the behavior, or the innovative idea at stake, many participants think that others in their working environment believe that they should not perform the behavior—i.e., they do not approve of the use of alternative methods (social normative pressure). This is expressed, for example, in lower publication chances, negative judgements by supervisors or failing the requirements that are imposed by funding agencies. Thus, the perception about a particular behavior—the use of alternative methods—is negatively influenced by the (perceived) judgment of others. Moreover, we found that many participants have a low self-efficacy, meaning that there is a perceived lack of behavioral control, i.e., their perceived ability to engage in the behavior at issue is low. Also, participants feel a lack of authority (in the sense of knowledge and skills, but also power) to initiate behavioral change. The existing subjective norms and perceived behavioral control, and the negative attitudes towards performing the behavior, lead to a lower behavioral intention, and, ultimately, a lower chance of the *performance* of the actual behavior.

Several participants mentioned there is a need for people of stature (belonging to the group of early adopters) to take the lead and break down perceived barriers. Early adopters serve as role models and have opinion leadership, and form the next group (after the innovators, in this case statisticians and methodologists) to adopt an innovative idea [20] (Fig 2). If early adopters would stand up, conveying a positive attitude towards the innovation, breaking down the described perceived barriers and facilitating the use of alternatives (for example by adjusting policy, guidelines and educational programs and making available financial resources for further training), this could positively affect the perceived social norms and self-efficacy of the early and late majority and ultimately laggards, which could ultimately lead to behavioral change among all stakeholders within the scientific community.

A strength of our study is that it is the first empirical study on views on the use of NHST, its alternatives and reasons for the prevailing use of NHST. Another strength is the method of coding which corresponds to the thematic approach from Braun & Clarke [47], which allows the researcher to move beyond just categorizing and coding the data, but also analyze how the codes are related to each other [47]. It provides a rich description of what is studied, linked to theory, but also generating new hypotheses. Moreover, two independent researchers coded all transcripts, which adds to the credibility of the study. All findings and the coding scheme were discussed by the two researchers, until consensus was reached. Also, interview results were further explored, enriched and validated by means of (mixed) focus groups. Important themes that emanated from the interviews, such as interdependency, perceptions on the scientific duty, perceived disadvantages of alternatives or the consequences of the current scientific climate, served as starting points and main subjects of the focus groups. This set-up provided more data, and more insight about the data and validation of the data. Lastly, the use of a theoretical framework [20,21] to develop the topic list, guide the interviews and focus groups, and guide their analysis is a strength as it provides structure to the analysis and substantiation of the results.

A limitation of this study is its sampling method. By using the network of members of the project group, and the fact that a relatively high proportion of those invited to participate refused because they thought they knew too little about the subject to be able to contribute, our sample was biased towards participants that are (somewhat) aware of the NHST debate. Our sample may also consist of people that are relatively critical towards the use of NHST, compared to the total population of researchers. It was not easy to include participants who were indifferent about or who were pro-NHST, as those were presumably less willing to make time and participate in this study. Even in our sample we found that the majority of our participants solely used NHST and perceived it as difficult if not impossible to change their behavior. These perceptions are thus probably even stronger in the target population. Another limitation, that is inherent to qualitative research, is the risk of interviewer bias. Respondents are unable, unwilling, or afraid to answer questions in good conscience, and instead provide socially desirable answers. In the context of our research, people are aware that, especially as a scientist, it does not look good to be conservative, complacent, or ignorant, or not to be open to innovation and new ideas. Therefore, some participants might have given a too favorable view of themselves. The interviewer bias can also take the other direction when values and expectations of the interviewer consciously or unconsciously influence the answers of the respondents. Although we have tried to be as neutral and objective as possible in asking questions and interpreting answers, we cannot rule out the chance that our views and opinions on the use of NHST have at times steered the respondents somewhat, potentially leading to the foregoing desirable answers.

Generalizability is a topic that is often debated in qualitative research methodology. Many researchers do not consider generalizability the purpose of qualitative research, but rather finding in-depth insights and explanations. However, this is an unjustified simplification, as generalizing of findings from qualitative research *is* possible. Three types of generalization in qualitative research are described: representational generalization (whether what is found in a sample can be generalized to the parent population of the sample), inferential generalization (whether findings from the study can be generalized to other settings), and theoretical generalization (where one draws theoretical statements from the findings of the study for more general application) [48]. The extent to which our results are generalizable is uncertain, as we used a theoretical sampling method, and our study was conducted exclusively in the Netherlands. We expect that the generic themes (reactivity, the scientific duty and the scientific climate) are applicable to academia in many countries across the world (inferential generalization). However, some elements, such as the Dutch educational system, will differ to a more or lesser extent from other countries (and thus can only be representationally generalized). In the Netherlands there is, for example, only one educational route after secondary school that has an academic orientation (scientific education, equivalent to the US university level education). This route consists of a bachelor’s program (typically 3 years), and a master’s program (typically 1, 2 or 3 years). Not every study program contains (compulsory) statistical courses, and statistical courses differ in depth and difficulty levels depending on the study program. Thus, not all the results will hold for other parts of the world, and further investigation is required.

Our findings demonstrate how perceived barriers to shift away from NHST set a high threshold for behavioral change and create a circle of interdependency. Behavioral change is a complex process. As ‘the stronger the intention to engage in a behavior, the more likely should be its performance’[21], further research on this subject should focus on how to influence the intention of behavior; i.e. which perceived barriers for the use of alternatives are most promising to break down in order to increase the intention for behavioral change. The present study shows that negative normative beliefs and a lack of perceived behavioral control regarding the innovation among individuals in the scientific system is a substantial problem. When social norms change in favor of the innovation, and control over the behavior increases, then the behavioral intention becomes a sufficient predictor of behavior [49]. An important follow-up question will therefore be: how can people be enthused and empowered, to ultimately take up the use of alternative methods instead of NHST? Answering this question can, in the long run, lead to the diffusion of the innovation through the scientific system as a whole.

## Conclusion

NHST has been the leading paradigm for many decades and is deeply rooted in our science system, despite longstanding criticism. The aim of this study was to gain insight as to why we continue to use NHST. Our findings have demonstrated how perceived barriers to make a shift away from NHST set a high threshold for actual behavioral change and create a circle of interdependency between stakeholders in the scientific system. Consequently, people find themselves in a state of reactivity, which limits behavioral change with respect to the use of NHST. The next step would be to get more insight into ways to effectively remove barriers and thereby increase the intention to take a step back from NHST. A paradigm shift within a couple of years is not realistic. However, we believe that by taking small steps, one at a time, it is possible to decrease the scientific community’s strong dependence on NHST and p-values.

## Supporting information

S1 Appendix (DOCX)
