# Supplementary material for: Why we habitually engage in null-hypothesis significance testing: A qualitative study
Source: PLoS One. 2021 Oct 15;16(10):e0258330. doi: 10.1371/journal.pone.0258330 (PMC8519469; doi:10.1371/journal.pone.0258330)

# **Appendix 1 Topic guide**

Interviews with lecturers and researchers

**Introduction**

*Aim:* *Introduction and context setting*, *gain trust, familiarize the participant*

- Introduction of researcher
- Study topic
- Explanations of the aims of the study
- Explanations of anonymity and member checking
- Explanation of recording
- Consent issues, withdrawal
- Any further questions?
- Continue?

**Background**

*Aim: To get the participant talking and to find out contextual information about his/her current circumstances*

- Current position
  - Job description
  - Position within department/faculty
- Research experience
- Teaching experience
- Current research

**Concept of NHST**

*Aim: To learn about the views on the foundation of drawing conclusions based on observed data*

- Beliefs about drawing conclusions (What is your perception of drawing ‘conclusions’ in your research? What is your definition of that word and what does it mean to you? How would you relate it to your observed data?)
  - Definition / meaning
  - Relation with regard to observed data
  - Difference between sample and population (What is your perception of the generalization of your conclusion?)
- Background of these beliefs (How did you come to those beliefs? How were you educated on this topic?)
- Frequentist statistics
  - Background, education (How were you educated? What is your perception of the foundation of frequentist statistics?)
  - Assumptions (perception and importance) (Do you check assumptions? Which one? What is your perception on the assumptions underlying frequentist statistics? What is your view on them? How do you apply them in research/teaching practice?)
- Hypotheses and conclusions
  - Meaning reject/not reject NH
  - Assumptions (perception and importance)
- Significance/P-value
  - Definition/meaning
  - Assumptions (perception and importance)
- Confidence intervals (Do you have any experience with this? Does it work for you?)
  - Definition/meaning
  - Assumptions (perception and importance)
- Effect sizes (Do you have any experience with this? Does it work for you?)
  - Definition/meaning
  - Assumptions (perception and importance)

**Use of NHST**

(*Aim: To explore how researchers are currently applying their perception of NHST)*

- Current use of NHST by the participant
- Statistical help (Do you do your statistical analyses yourself?)
  - Testing
  - Statistical power
- Attractiveness (What do you like about using it? Why do you prefer it?)
- Drawbacks (What do you dislike about using it? Why?)
- Familiarity with NHST (How familiar is using it to you?)

Environment

- Current policy and atmosphere at department or institution concerning NHST (Is everyone using it? Is there a guideline?)
- Colleagues using NHST or other methods
- Conversation about NHST with colleagues (Is this topic open for discussion? If yes, is this organized and ? How is this organized and supported by management?)

**Future of NHST**

*(Aim: To learn the views about a possible switch to an alternative for NHST)*

- Knowledge
  - Knowledge of current debate about NHST
  - Knowledge of the problems associated with NHST
- Alternatives (NOT listing them, only asking for current knowledge and then about views in general)
  - Knowledge
  - Views (How do you feel about these alternatives?)
- Stop using NHST (How would you feel about switching to a different method? Why, can you elaborate on that?)
- Innovation
  - Perception and expectation (How would you feel about a possible *innovation* to implement an alternative for NHST? How would you regard such an innovation? What would you expect, what should it be about?)
  - Content (What do you think is important to include in such an innovation? What should be left out?)
  - Implementation (If you were to implement such an innovation in your own research/teaching, what strategy would you use? How would you do it?)
  - Resources (If you were to implement such an innovation in your own research/teaching, what resources would you require (Schooling, assistance etc)?)
  - Time (If we you were to implement such an innovation in your own research/teaching, what timeline would you use? At once, grace period?)
  - Impact
    - Own research (What do you think would be the impact on your own research?)
    - Scientific research in general (What would you think the impact would be on scientific research in general (Outcomes, publications, integrity)?)

**Rounding up**

*(Aim: Winding down; switching to ‘daily life’: possibility to address other topics that the participant may think of)*

- Any other thoughts about NHST
- Other thoughts about this project

**Concluding**

*(Aim: to reiterate ethical issues, promises and procedure, closing)*

- Thanks
- Repeat anonymity and member check
- Contact information
- Further steps in the research

Interviews with editors and representatives from funding agencies

**Introduction**

*Aim:* *Introduction and context setting*, *gain trust, familiarize the participant*

- Introduction of researcher
- Study topic
- Explanations of the aims of the study
- Explanations of anonymity and member checking
- Explanation of recording
- Consent issues, withdrawal
- Any further questions?
- Continue?

**Background**

*Aim: To get the participant talking and to find out contextual information about his/her current circumstances*

- Current position
  - Job description
  - Position within organization
- Study background
- Research experience
- Teaching experience

**Concept of NHST**

*Aim: To learn about the views on the foundation of drawing conclusions based on observed data*

*Meer nadruk op: wat voor rol spelt NHST voor jou/voor je werk*

*hier wat korter/minder diep op in gaan*

- Beliefs about drawing conclusions (What is your perception of drawing ‘conclusions’?)What is your definition of that word and what does it mean to you?
  - Definition / meaning
  - Relation with regard to observed data
  - Difference between sample and population (What is your perception of the generalization of the conclusion?)
- Background of these beliefs (How did you come to those beliefs? How were you educated on this topic?)
- Frequentist statistics
  - Background, education (How were you educated? What is your perception of the foundation of frequentist statistics?)
  - Assumptions (perception and importance) (What is your perception on the assumptions underlying frequentist statistics? What is your view on them?)
- Hypotheses and conclusions
  - Meaning reject/not reject NH
  - Assumptions (perception and importance)
- Significance/P-value
  - Definition/meaning
  - Assumptions (perception and importance)
- Confidence intervals (Do you have any experience with this? Does it work for you?)
  - Definition/meaning
  - Assumptions (perception and importance)
- Effect sizes (Do you have any experience with this? Does it work for you?)
  - Definition/meaning
  - Assumptions (perception and importance)

**Use of NHST**

*Focus on: what does NHST mean to you and what role does it play in your work?*

(*Aim: To explore how researchers are currently applying their perception of NHST)*

- Familiarity with NHST (How familiar is using it to you?)
- Attractiveness (What do you like about it? Why do you prefer it?)
- Drawbacks (What do you dislike about it? Why?)
- **Policy concerning NHST within organization**
- What is the current policy with regard to statistics and methodology when judging research proposals (guidelines, requirements)? Which aspects do you consider?
- Does a research proposal require a statistics and methodology section to be accepted?
- Who determines the guidelines/requirements (with regard to statistics)? Why do you require and maintain this policy/these guidelines? How were these guidelines established (where does input come from?)?
- Who judges the statistical part of a research (proposal)? Is there mutual consultation about the statistical section? Do you have sparring partners when evaluating the statistical section?
- Is the application of NHST a requirement?
- What is the atmosphere within the organisation concerning NHST
- Colleagues preferring NHST or other methods?
- Conversation about NHST with colleagues (Is this topic open for discussion? If yes, is this organized? How is this organized and supported by management?)

**Future of NHST**

*(Aim: To learn the views about a possible switch to an alternative for NHST)*

- Knowledge
  - Knowledge of current debate about NHST
  - Knowledge of the problems associated with NHST
- Alternatives (NOT listing them, only asking for current knowledge and then about views in general)
  - Knowledge
  - Views (How do you feel about these alternatives?)
- Stop using NHST (How would you feel about switching to a different method? Why, can you elaborate on that?)
- Innovation
  - Perception and expectation (How would you feel about a possible *innovation* to implement an alternative for NHST? How would you regard such an innovation? What would you expect, what should it be about?)
  - Content (What do you think is important to include in such an innovation? What should be left out?)
  - Implementation (If you were to implement such an innovation in your own policy, what strategy would you use? How would you do it?)
  - Resources (If you were to implement such an innovation in your own policy, what resources would you require (Schooling, assistance etc)?)
  - Time (If we you were to implement such an innovation in your own research/teaching, what timeline would you use? At once, grace period?)
  - Impact
    - Scientific research in general (What would you think the impact would be on scientific research in general (Outcomes, publications, integrity)?)

**Rounding up**

*(Aim: Winding down; switching to ‘daily life’: possibility to address other topics that the participant may think of)*

- Any other thoughts about NHST
- Other thoughts about this project

**Concluding**

*(Aim: to reiterate ethical issues, promises and procedure, closing)*

- Thanks
- Repeat anonymity and member check
- Contact information
- Further steps in the research

# **Appendix 2: Complete coding tree**


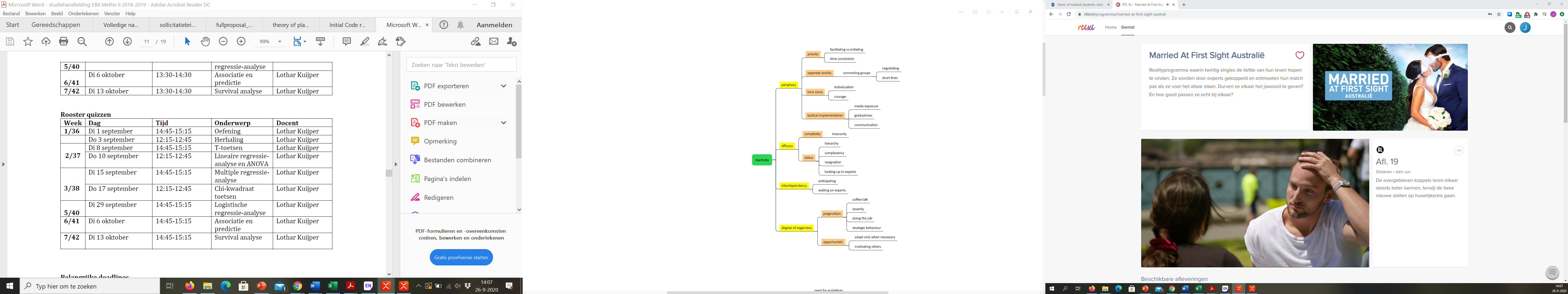


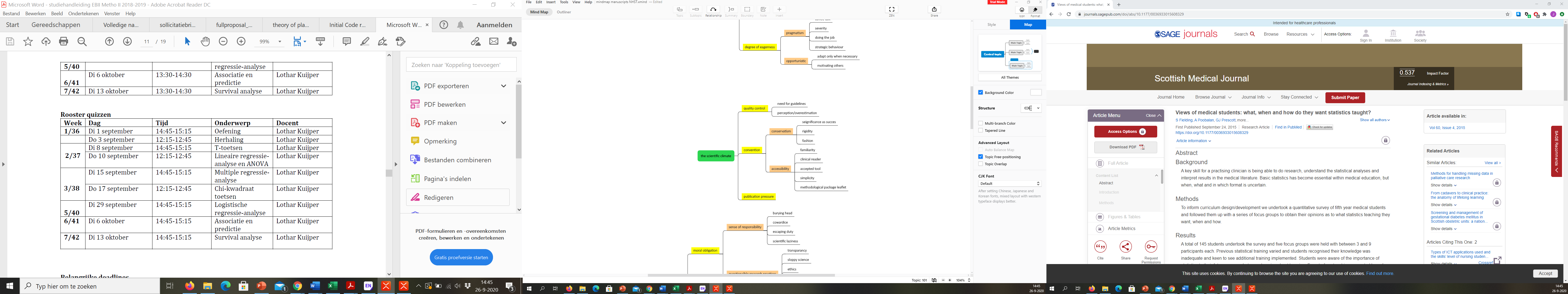


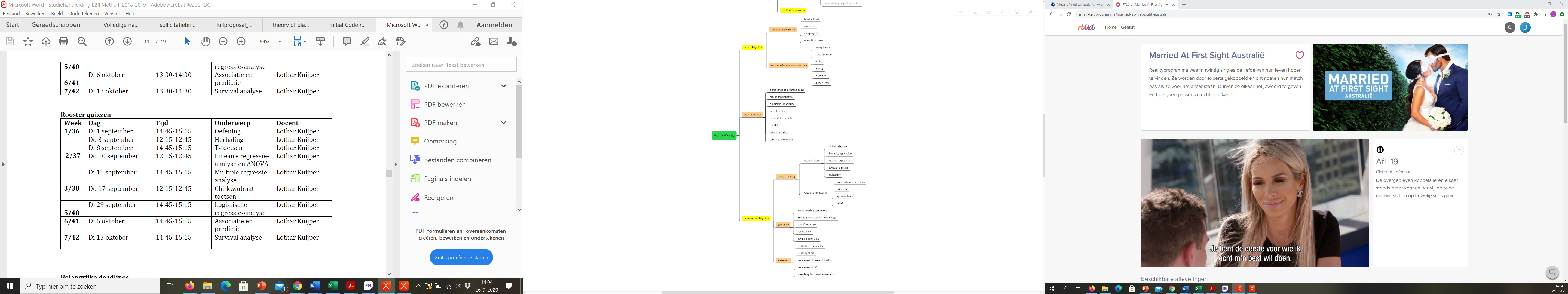

Supplement: S1 Appendix — (DOCX) [file pone.0258330.s001.docx]
